# Baculum shape complexity correlates to metrics of post‐copulatory sexual selection in Musteloidea

**DOI:** 10.1002/jmor.21572

**Published:** 2023-03-01

**Authors:** Emma Clear, Robyn Grant, James Gardiner, Charlotte Brassey

**Affiliations:** ^1^ Faculty of Science and Engineering Manchester Metropolitan University Manchester UK; ^2^ Institute of Life Course and Medical Sciences The University of Liverpool Liverpool UK

**Keywords:** baculum, evolution, genitalia, Musteloidea, sexual selection, shape complexity

## Abstract

The penis bone, or baculum, is present in many orders of mammals, although its function is still relatively unknown, mainly due to the challenges with studying the baculum in vivo. Suggested functions include increasing vaginal friction, prolonging intromission and inducing ovulation. Since it is difficult to study baculum function directly, functional morphology can give important insights. Shape complexity techniques, in particular, are likely to offer a useful metric of baculum morphology, especially since finding homologous landmarks on such a structure is challenging. This study focuses on measuring baculum shape complexity in the Musteloidea—a large superfamily spanning a range of body sizes with well‐developed, qualitatively diverse bacula. We compared two shape complexity metrics—alpha shapes and ariaDNE and conducted analyses over a range of six different coefficients, or bandwidths, in 32 species of Musteloidea. Overall, we found that shape complexity, especially at the baculum distal tip, is associated with intromission duration using both metrics. These complexities can include hooks, bifurcations and other additional projections. In addition, alpha shapes complexity was also associated with relative testes mass. These results suggest that post‐copulatory mechanisms of sexual selection are probably driving the evolution of more complex‐shaped bacula tips in Musteloidea and are likely to be especially involved in increasing intromission duration during copulation.

## Graphical abstract

We apply three‐dimensional complexity metrics—alpha shapes and ariaDNE—to Musteloidea baculum. Increased complexity is associated with morphological differences in the baculum distal tip. Complexity scores correlate with intromission duration and relative testes mass, suggesting post‐copulatory sexual selection may be driving the evolution of baculum shape in this group.

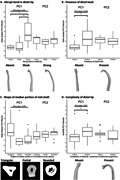

## 1 INTRODUCTION

The mammal os penis, or baculum, is a bone located in the male glans and has been documented in species belonging to at least nine mammalian orders, including Afrosoricida, Carnivora, Chiroptera, Dermoptera, Erinaceomorpha, Primates, Rodentia, Soricomorpha and Lagomorpha (Brindle & Opie, 2016). The evolution of the baculum is subject to multiple gains and losses and not considered a homologous structure (Schultz et al., 2016). Alongside variation in presence and absence, there is variability in the shape of the baculum, with features including ‘hooks’, ‘scoops’, urethral grooves and other distinct characteristics often used in taxonomy to determine phylogenetic relationships (Hooper & Musser, 1964; Pocock, 1918). Historically, it was argued that this variability may represent a by‐product of species divergence (Best & Schnell, 1974; Burt, 1936), but there is now strong evidence to suggest that the baculum is subject to direct sexual selection (André et al., 2021; Hosken & Stockley, 2004; Stockley et al., 2013). However, as yet, there is no clear consensus on the function of the baculum. Three key theories have been suggested, including: (i) the ‘vaginal friction hypothesis’ (Long & Frank, 1968), where the additional rigidity of the bacula facilitates intromission, specifically in taxa presenting high levels of sexual size dimorphism (SSD) or where the act of mounting occurs before erection (Lariviere & Ferguson, 2002); (ii) the ‘prolonged intromission hypothesis’ (Ewer, 1973), in which the bacula enables successful sperm deposition by preventing the urethra from becoming occluded during long periods of intromission and (iii) the ‘induced ovulation hypothesis’ (Greenwald, 1956), whereby the additional stiffness and/or tip shape provided by the baculum stimulates the reproductive tract of the female, triggering ovulation and increasing the likelihood of fertilisation. Support for all three hypotheses has been only sporadically found across mammals (André et al., 2021; Brindle & Opie, 2016; Dixson et al., 2004). Partly this is due to the challenges and invasiveness associated with testing these hypotheses in vivo. We can, instead, investigate the functional morphology of the baculum (as per Brassey et al., 2020), and make associations between its form and function.

However, characterising baculum form is also challenging. Previous studies often assume that the mechanical function of bacula can be inferred from simple, linear metrics—usually baculum length and diameter (Dixson, 1987; Long & Frank, 1968). Although it is unlikely that simple beam models such as these are adequate (Brassey et al., 2013), especially in capturing the mechanical implications of characteristics such as hooks or grooves. Other studies have conducted simple qualitative scoring of morphological characters (Baryshnikov et al., 2003; Kankiliç et al., 2014), although these lack quantification, since they are usually only scored on a one or two‐point scale. The development of geometric morphometrics (GMM) and the application of anatomical landmarking (Rohlf & Marcus, 1993) has the potential to identify changes in shape in homologous structures (Drake et al., 2017; Murta‐Fonseca et al., 2019; Romaniuk, 2018); however, its application to mammalian bacula is problematic given the lack of discrete homologous landmarks.

An analysis of shape *complexity* could address the challenges of quantifying bacula form. Complexity can be broadly defined as the number of ‘parts’ comprising the topography of a structure, with high complexity having a high number of ‘primitive’ shapes (cylinder, spheres, cubes etc.; Gardiner et al., 2018). Many techniques have been developed to rapidly quantify three‐dimensional (3D) shape complexity in anatomical structures, including orientation patch count (Evans et al., 2007), relief index (Boyer, 2008), Dirichlet normal energy (DNE) (Bunn et al., 2011) and ariaDNE (Shan et al., 2019), but few have been applied to genital structures. In invertebrates, the morphology of insect genitalia has been investigated using outline‐based Elliptical Fourier Analyses and dissection indices (perimeter:area ratio) (Rowe & Arnqvist, 2011; Simmons & Fitzpatrick, 2019; Song & Bucheli, 2010). In vertebrates, Brassey et al. (2020) explored baculum morphological diversity in carnivoran genitalia using a novel alpha shapes complexity method (Gardiner et al., 2018). Their results suggested that post‐copulatory sexual selection pressures, such as prolonged intromission duration and sperm competition, are likely to be associated with complex morphology at the distal tip of the bacula in carnivores. However, it is not yet clear which elements of tip morphology are driving the results of this metric. Indeed, shape complexity techniques tend to assess complexity across an entire structure and lack the specificity to differentiate between characters. Therefore, a combination of both qualitative and quantitative analysis is necessary to understand which morphological features contribute to overall complexity scores. In line with the work of Brassey et al. (2020), we may expect to find that high complexity scores of bacula shape are associated with structures at the baculum tip, more so than on the shaft or base. These may include ‘hooks’, bifurcations and other additional tip projections.

Here, we quantify 3D baculum shape complexity across a closely related group of carnivoran mammals, the Musteloidea. This closely related superfamily spans a range of body sizes and possess well‐developed, qualitatively diverse bacula (Kitchener et al., 2017; Law et al., 2017). The largest musteloid family, Mustelidae, present increased levels of SSD compared to other carnivoran groups (Law, 2019) and many engage in post‐copulatory mate guarding behaviours associated with prolonged intromission duration (Dixson, 2021), making them an interesting and appropriate focus for this investigation. We present a methodological comparison of two shape complexity metrics—ariaDNE and alpha shapes. Historically, the two metrics have been developed for different applications; DNE was originally used to examine crown complexity of mammalian teeth, whereas alpha shapes was initially applied to long‐bone structures. The underlying mathematics of the two complexity algorithms are different, and previous comparisons of shape complexity metrics have found resulting complexity scores to be highly divergent depending on the method used (Arslan et al., 2022; Gardiner et al., 2018). On the basis of this literature, we might expect ariaDNE to better resolve fine scale features and assign higher complexity values to bacula with extreme distal tip morphologies, such as acute hooks and additional projections. In contrast, alpha shapes may be more sensitive to gross morphology, attributing high complexity values to those bacula with characteristics such as bends in the distal tip and long urethral grooves.

We correlate these two complexity metrics to pre‐ and post‐copulatory selection pressures to identify the likely mechanisms of sexual selection. We may expect to find that species exhibiting pre‐copulatory mechanisms, with high levels of SSD, will possess simple, rod‐like bacula. There may also be a positive relationship between species displaying post‐copulatory selection traits, including extended periods of intromission or increased testes mass, with complex baculum morphology across the group. Finally, we compare 3D shape complexity metrics against a qualitative scoring method to further explore the anatomical characters underlying complexity scores of the baculum.

## 2 MATERIALS AND METHODS

### 2.1 Specimens

Specimens of Musteloidea bacula (*n* = 32) were loaned from osteological collections of the National Museum of Scotland, Edinburgh (NMS) and the Natural History Museum, London (NHM). Samples represent 38% of the total extant Musteloidea species (Law et al., 2017), covering the families Ailuridae (*n* = 1), Mustelidae (*n* = 28) and Procyonidae (*n* = 3). Individuals that were obviously juvenile or possessed pathologies were excluded from analysis. Complete list of specimens and corresponding life history information can be found in the Supporting Information.

### 2.2 Data collection

Specimens were scanned at the Manchester X‐Ray Imaging Facility in a 320/225 Nikon X‐Tek Custom Bay. Specimens loaned from the NHM were micro‐computerised tomography (CT) scanned at the museum's Imaging and Analysis Centre, using a Nikon Metrology HMXST 225 scanner. Average resolution for data set was 34 µm. The 32‐bit.raw CT files were converted to binary data using Fiji and exported as 8‐bit.raw files.

Both male and female body masses were predominantly assigned from existing published literature (*n* = 32; see Supporting Information). In some instances, testes mass included both testes and epididymis mass (*n* = 5), representing the upper limit of the typical testes mass size for the species. In the absence of alternative data, testes volume/linear dimensions were taken as a representative of testes mass, under the assumption of an average testes' density of 0.81 kg m^−13^ (*n* = 7) as per Brassey et al. (2020). Likewise, intromission durations were also collated from the literature. Any life history data that was unavailable are listed as NA in Supporting Information.

### 2.3 Shape complexity methods

To quantify 3D topographic complexity of the baculum, two methodologies were applied to the data: ariaDNE (Shan et al., 2019) and alpha shapes (Gardiner et al., 2018). Both metrics can be applied to 3D mesh models allowing shape analysis of the whole bone, and each have the capacity to explore how taxa differ across a scale of shape complexity (from fine scale features to coarse gross morphology). Both complexity methodologies were applied using MATLAB (The Mathworks Inc.).

#### 2.3.1 Alpha shapes

The alpha shapes protocol is provided in detail in Gardiner et al. (2018). In summary, the 8‐bit.raw CT data were converted from voxels to 3D point clouds. These were then decimated to 100,000 points each and scaled to remove the effect of size. Alpha shapes were then applied to the data in MATLAB at 6 ‘refinement coefficients’ (see Supporting Information) spread over a logarithmic scale to assess bacula complexity. The coefficients were chosen to achieve two goals. First, to cover a broad range of morphological features, with the lowest refinement coefficients resolving fine morphological details (such as surface textures, small pits/grooves etc.) and higher refinement coefficients resolving larger, gross anatomy (such as curvature along the baculum length). Outside of our selected coefficient range, fits were either too fine resulting in the alpha shape ‘breaking down’ into multiple volumes that bear no resemblance to the underlying anatomy or too large with fits all identical to a convex hull. Second, six fits were chosen, to strike a balance between capturing the changes that occur with different refinement coefficients but avoiding oversampling. Balancing the number of samples (species) with variables (refinement coefficients or ariaDNE bandwidths) is also recommended for analysis such as principal component analysis (PCA).

#### 2.3.2 AriaDNE

The ariaDNE analysis followed the methods by Shan et al. (2019). AriaDNE is an updated metric developed from the original, DNE (Bunn et al., 2011). Compared to DNE, ariaDNE is less sensitive to a greater range of mesh preparation protocols, limiting the negative effects associated with differing triangle counts, noise and smoothing (Shan et al., 2019). Briefly, it assesses local geometric information across each face of a 3D mesh and measures the degree to which a surface deviates from a planar (zero). It calculates the sum for the whole bone, with higher values indicative of more complex shapes. Rather than the .raw files, ariaDNE is applied to isosurface mesh models. Using the 8‐bit.raw files, 3D isosurface meshes were generated using standard Drishti protocol (Limaye, 2012). Files are then exported via a mesh generator package as .ply files. To maximise comparability of specimens and improve consistency in surface detail, the 3D mesh models were decimated in size to ~30 k triangles using Geomagic Studio (3D Systems). AriaDNE was calculated for individual bacula by applying the algorithm in MATLAB (Shan et al., 2019) to each of the .ply isosurface mesh files. The ariaDNE results presented herein were taken at a range of 6 equally spaced parameters (termed ‘bandwidths’) as suggested by Shan et al. (2019) to explore how baculum complexity differs at multiple scales (0.02, 0.04, 0.06, 0.08, 0.1 and 0.12). Deciding on the appropriate bandwidth depends upon the study at hand. If set too low, results will be highly sensitive to trivial features, whereas if set too high, the approximation will become non‐local and risks overlooking relevant morphological details (Shan et al., 2019).

### 2.4 Statistical analysis

We conducted a PCA performed on a correlation matrix incorporating all bacula shape complexity results at each of the six refinement coefficients/bandwidths for both alpha shapes and ariaDNE using the “prcomp” function of R (v4.1.3; R Core Team, 2021) to summarise the key features captured. The two principal components describing the most variation in the data set for both alpha shapes (84.65% in total) and ariaDNE (97.59% in total), were analysed to address the following two questions:

#### 2.4.1 Which mechanisms of sexual selection are associated with baculum shape complexity?

The relationships between baculum shape complexity and mechanisms of sexual selection were first investigated using separate linear regression analyses. To investigate the influence of pre‐copulatory selection mechanisms against PCA complexity results, SSD was included as a predictor variable. SSD was calculated using the formula male mass/female mass (as per Fitzpatrick et al., 2012). To assess the impact of post‐copulatory sexual selection on baculum shape complexity, the influence of both intromission duration and intensity of sperm competition was examined against the PCA complexity results. Both, male body mass and testes mass, were added as independent variables to the model using the formula male mass + testes mass, this provided a measure of residual testes mass, a commonly used proxy to indicate strength of sperm competition (Fitzpatrick et al., 2012). All life history data were log‐transformed.

Following the initial regression models between baculum complexity PCA scores (alpha shapes PC1 and PC2, and ariaDNE PC1 and PC2) and the three proxies for mechanisms of sexual selection (SSD, intromission duration, testes mass), the analyses were repeated using phylogenetically controlled regressions to account for nonindependence of data. All statistical analyses were completed in R (v4.1.3; R Core Team, 2021). These were conducted using the caper package (Orme et al., 2013) in R, with a *λ* correlation structure (Pagel, 1999). Pagel's *λ* offers a measure of strength of phylogenetic signal, normally varying between 0 (indicating total lack of phylogenetic signal) and 1 (indicating fit with Brownian motion). Here, *λ* was set to an estimate of maximised likelihood and also explored when set at 1. Phylogenetic relationships between all species in the data set were represented using a sample of 10,000 trees downloaded from Vertlife.org (Upham et al., 2019), a consensus tree was then created using the TreeAnnotator function within the BEAST programme (v1.10.4; Suchard et al., 2018; see Supporting Information).

#### 2.4.2 Which features of the baculum are associated with shape complexity?

To understand the contributions of discrete osteological features to the calculated complexity scores, the PCA models (i.e., PCA1, PCA2 etc.) of both alpha shapes and ariaDNE, which significantly correlated to any one of the three proxies (SSD, intromission duration, testes mass) were compared to a qualitative scoring metric. Each baculum was scored across nine character states sourced from Baryshnikov et al. (2003). Two character states relate to the head of the bacula, two for the bacula shaft and five focus on the bacula distal tip (Table 1). These characters were given a score of either 0, 1 (i.e., presence/absence) or a 0, 1, 2 with low numbers attributing to absent/weakly developed features and higher numbers indicating prominent development. Scoring was completed by the main author. This process was also conducted by five independent researchers to assess the repeatability of the approach (see Supporting Information: Appendix 1). As most of the data were not normally distributed and the total data set was relatively small at only 32 samples, non‐parametric testes were applied. Kruskal–Wallis and pairwise Wilcoxon statistical tests were used to investigate the difference between scores across the qualitative results.

**Table 1:** Table identifying character states for nine morphological features relating to the head, shaft and tip of the bacula used for qualitative analysis.

I.D.	Character	Score I.D.		
*Relating to bacula head*				
H1	Pronounced head	0 = absent	1 = weak/slightly pronounced	2 = strong/well marked
H2	Opening on head	0 = absent	1 = present	
*Relating to bacula shaft*				
S1	Shape of median portion of mid‐shaft in cross section	0 = triangular, dorsal crest well pronounced	1 = rounded/triangular, dorsal crest not pronounced	2 = rounded, dorsal crest absent
S2	Absence of urethral groove	0 = absent	1 = short, only present distally	2 = long
*Relating to bacula distal tip*				
T1	Abrupt upward bend in distal tip	0 = absent	1 = weakly present	2 = strongly present
T2	Presence of distal hook	0 = absent	1 = present	
T3	Subdivision of distal tip in ventral plane	0 = absent	1 = present	
T4	Shape of distal tip	0 = symmetrical	1 = asymmetrical	
T5	Complexity of distal tip	0 = additional projections absent	1 = additional projections present	

*Note*: Modified from Baryshnikov et al. (2003).

## 3 RESULTS

### 3.1 PCA comparisons of alpha shapes and ariaDNE

**Figure 1:**
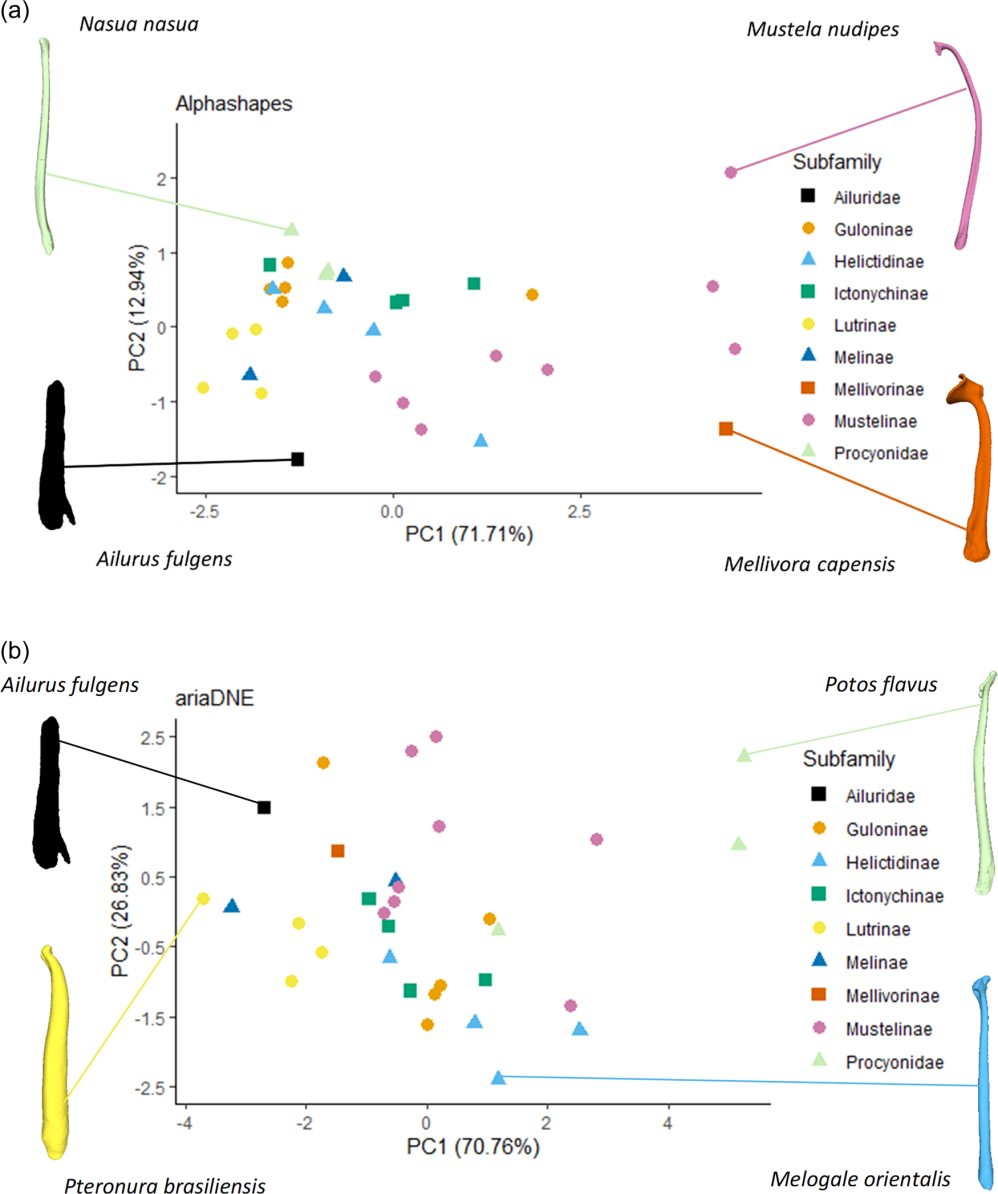
Scatter plots of PCA results for both complexity metrics (a) Alpha shapes PCA results (PC1 and PC2) for musteloid species identified by subfamily. Representative species displayed as 3D mesh and labelled (bones not to scale). (b) AriaDNE PCA results (PC1 and PC2) for musteloid species identified by subfamily. Representative species displayed as 3D mesh (bones not to scale). PCA, principal component analysis; 3D, three‐dimensional.

PCA for both ariaDNE and alpha shapes show some clustering according to subfamily, but still display overlap across species. Alpha shapes PC1 (Figure 1a) accounts for 72% of total variation in the data set, while PC2 describes 13%. Species with high alpha shapes PC1 scores, *Mellivora capensis* and three from the family Mustela, all feature prominent curvature of the baculum bone, especially a bend in the distal tip (Figure 1a see meshes labelled *M. capensis* and *Mustela nudipes*). The family Guloninae shows some clustering within the group; however the one outlier, *Martes flavigula*, features a bend at the distal tip and scores highly in alpha shapes PC1. Species from the family Lutrinae held the lowest alpha shape PC1 scores, and all have simplistic, club‐like form (Figure 1a). Alpha shapes PC2 appears to describe bacula aspect ratio, with long, slim bacula from the family Procyonidae having high PC2 scores, and thicker bacula like that of *M. capensis* and *A. fulgens* having lower PC2 scores (Figure 1a see examples labelled *Nasua nasua* and *A. fulgens*).

AriaDNE PC1 (Figure 1b) accounted for 71% of total variation and appears to separate species on the basis of baculum tip ornamentation. For example, *M. nudipes, Potos flavus* and *N. nasua* have the highest ariaDNE PC1 scores and have a prominent bend at the distal tip, while *A. fulgens* and *Pteronura brasiliensis* have the lowest PC1 scores and possess simplistic, club‐like bacula (Figure 1b see differences in morphology of *A. fulgens* and *P. flavus*). AriaDNE PC2 accounts for 27% of variation but overall variation is unclear. Species with the largest ariaDNE PC2 scores, *Mustela sibirica*, *Mustela kathiah* and *P. flavus* possess urethral grooves that run the length of the bone and bends in mid‐section and at the distal tip (Figure 1b), while *Melogale orientalis* held the lowest ariaDNE PC2 score, with a bacula featuring a rounded shaft and clubbed head (Figure 1b).

#### 3.1.1 Which mechanisms of sexual selection influence baculum shape complexity?

**Figure 2:**
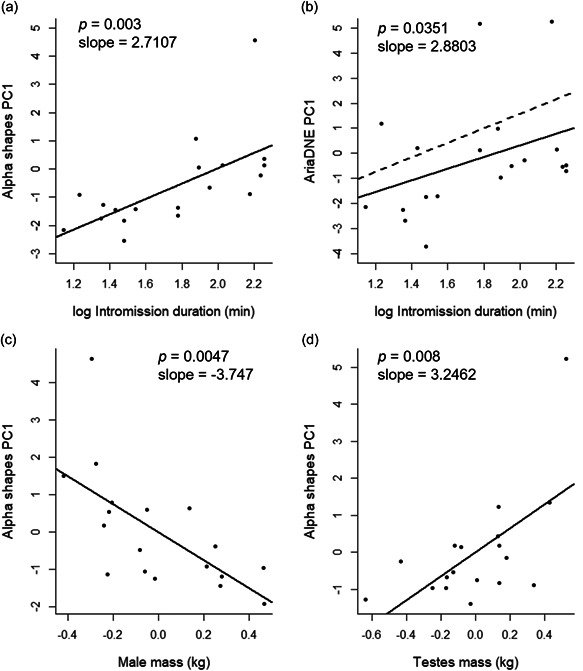
Scatter plots depicting the relationship between shape complexity metric and sexual selection mechanism. (a) Alpha shapes PC1 and intromission duration. (b) AriaDNE PC1 and intromission duration. (c) residual alpha shapes PC1 and residual body mass. (d) residual alpha shapes PC1 and residual testes mass. Solid line indicates linear model best fit, dotted line indicates PGLS best fit. PGLS not plotted in a, c and d as *λ* values were identical.

Results for both linear models and (phylogenetic generalized least square regression [PGLS]; *λ* = maximum likelihood estimate and *λ* = 1) are displayed in Table 2. The regression models did not identify a significant relationship between SSD and any of the shape complexity results from either ariaDNE or alpha shapes PCA. A significant positive relationship was found between alpha shapes PC1 and intromission duration following both, phylogenetically uncorrected linear regression (Figure 2a; *n* = 20, adjusted *R* ^2^ = .3782, *F*(1, 16) =11.95, *p* = .003) and PGLS (*λ* = 0.001, adjusted *R* ^2^ = .3782, *F*(1, 16) = 11.95, *p* = .003). AriaDNE PC1 had no significant relationship with intromission duration during linear modelling (adjusted *R* ^2^ = .0922, *F*(1, 16) = 2.828, *p* = .1109) but displayed a significant relationship with PGLS when *λ* was estimated (*λ* = 1; Figure 2b; adjusted *R* ^2^ = .1908, *F*(1, 16) = 5.244, *p* = .0351). A significant positive relationship was found between relative testes mass and alpha shapes PC1 (Figure 2c,d; *n* = 18, adjusted *R* ^2^ = .3491, *F*(2, 15) = 5.559, body mass *p* = .0047, testes mass *p* = .008). This significance was upheld when accounting for phylogenetic nonindependence (*λ* = 0.001, adjusted *R* ^2^ = .3491, *F*(2, 15) = 1.53, body mass *p* = .0047, testes mass *p* = .008).

**Table 2:** Table displaying results of phylogenetically uncorrected linear model, PGLS (*λ* = maximum likelihood estimate) and PGLS (*λ* = 1) results.

		Phylogenetically uncorrected linear model (*λ* = 0)			PGLS (*λ* = ML)				PGLS (*λ* = 1)		
Proxy	Formula	*p* Value	Slope	Adjusted *r* ^2^	*p* Value	Slope	Adjusted *r* ^2^	*λ*	*p* Value	Slope	Adjusted *r* ^2^
Intromission	Alpha PC1	.003*	2.7079	.3782	.003*	2.7107	.3782	0	.4782	1.6287	−.027
	Alpha PC2	.832	−0.1186	−.0559	.1962	0.1298	−.0522	0.877	.8754	0.1191	−.0573
	AriaDNE PC1	.1109	2.3072	.0922	.0351*	2.8803	.1908	1	.0351*	2.8803	.1908
	AriaDNE PC2	.3057	0.7857	.007	.293	0.7909	.0098	0	.8997	0.1796	−.0578
Sexual size dimorphism (SSD)	Alpha PC1	.0873	5.3138	.0712	.0873	5.3138	.0712	0	.3847	1.6321	−.0078
	Alpha PC2	.8433	−0.2889	−.036	.6781	0.4782	−.0303	0.8	.1457	1.160	.0425
	AriaDNE PC1	.9512	−0.2053	−.0369	.5629	0.7738	−.02402	1	.5629	0.7738	−.024
	AriaDNE PC2	.3458	1.8144	−.0028	.1172	2.140	.0547	0.864	.0538	1.9139	.0987
Testes mass	Alpha PC1	M 0.0047* T 0.008* 0.01562*	M −3.7470 T 3.2463	.3491	M 0.0047* T 0.008* 0.01562*	M −3.747 T 3.2462	.3491	0	M 0.1010 T 0.2073 0.2485	M −1.9226 T 1.0540	.0587
	Alpha PC2	M 0.337 T 0.331 0.5973	M 0.8577 T −0.8163	−.0581	M 0.7654 T 0.7293 0.9396	M 0.2171 T −0.1994	−.124	0.947	M 0.9589 T 0.9876 0.9978	M 0.0382 T ‐0.0084	−.133
	AriaDNE PC1	M 0.211 T 0.359 0.4156	M −2.146 T 1.4592	−.0081	M 0.1974 T 0.3924 0.4095	M −1.5128 T 0.7179	−.0062	1	M 0.1974 T 0.3924 0.4095	M −1.5128 T 0.7179	−.0061
	AriaDNE PC2	M 0.387 T 0.252 0.4882	M −0.8833 T 1.1100	−.03001	M 0.387 T 0.2523 0.4882	M −0.8833 T 1.11	−.03001	0	M 0.5628 T 0.5328 0.809	M −0.5584 T 0.4378	−.1018

*Note*: All significant results are indicated by an asterisk.

#### 3.1.2 Which features of the baculum are associated with shape complexity?

**Figure 3:**
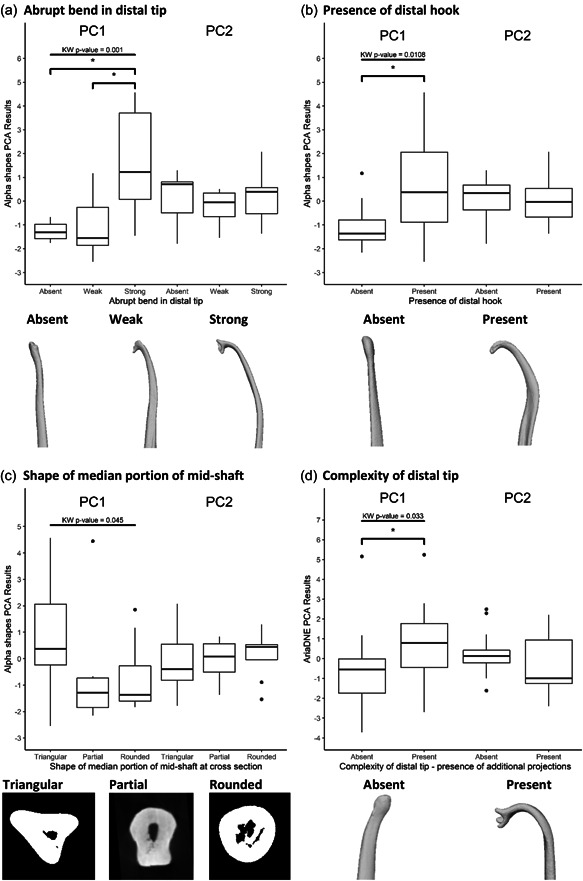
Box plots depicting alpha shapes and ariaDNE PCA results, split between morphological character scores. Results indicate that both alpha shapes PC1 and ariaDNE PC1 are positively correlated with post‐copulatory selection mechanisms. Further statistical analyses on these data found significant associations between alpha shapes PC1 and ariaDNE PC1 and four baculum characters. Alpha shapes PC1 was associated with (a) abrupt bend in distal tip (representative species in order shown—*Meles meles*, *Mustela putorius*, *Mustela nudipes*), (b) presence of distal hook (representative species in order shown—*Lyncodon patagonicus*, *Mustela kathiah*) and (c) and shape of median portion at cross section (representative species in order shown—*Mellivora capensis*, *Mustela lutreola*, *Potos flavus*). AriaDNE PC1 was associated with (d) Complexity at the distal tip (representative species in order shown—*Enhydra lutris*, *Martes flavigula*). Lines indicate significant results for Kruskall–Wallis test. Significant statistical results found between scores using a pairwise—Wilcoxon and results are indicated via asterisk. Renderings below each boxplot exemplify typical bacula for the result. Bacula mesh models are depicted in the lateral plane. (c) Depicts the median slices taken from the .raw data. PCA, principal component analysis.

Two features relating to the baculum distal tip were significantly associated with alpha shapes PC1, including the ‘abrupt bend in distal tip’ (Figure 3a, Kruskal–Wallis, *H*(2) = 13.877, *p* = .001) and the ‘presence of distal hook’ (Figure 3b, Kruskal–Wallis, *H*(1) = 6.4973, *p* = .0108). Pairwise comparisons indicated that the ‘abrupt bend in distal tip’ was significantly different in alpha shapes PC1 between scores 0–1 (*p* = .009) and 0–2 (*p* = .0012). The ‘presence of distal hook’ also significantly differed between scores 0–1 (*p* = .001) in alpha shapes PC1.

We also found a significant association between one of the shaft features, ‘shape of median portion of cross section’ with alpha shapes PC1 (Figure 3c, Kruskal–Wallis, *H*(2) = 6.2126, *p* = .045), but pairwise comparisons showed no significant differences between scores 0 and 2. AriaDNE PC1 was significantly associated with one feature relating to the tip of the baculum, ‘the complexity of the distal tip’ (Figure 3d, Kruskal–Wallis, *H*(1) = 4.5522, *p* = .033) and pairwise Wilcoxon comparisons found significant relationship between scores 0 and 1 (*p* = .033), which accounts for the presence of additional projections such as scoops or a prong‐like characters.

## 4 DISCUSSION

A significant relationship was found between alpha shapes PC1 and two metrics of post‐copulatory sexual selection: intromission duration and relative testes mass. Morphological character analysis revealed that these results were associated with shape complexity at the baculum distal tip and shaft. AriaDNE PC1 results were also significantly related to intromission duration and associated with complexity of the distal tip, namely the presence of ‘additional projections’. These results suggest that post‐copulatory mechanisms of sexual selection are likely to be driving the evolution of more complex shaped bacula tips in Musteloidea.

Alpha shapes PC1 described the majority of variation and is positively loaded towards regional shape complexity at the bacula distal tip, namely curvature of the bone and presence of a hook. These findings are in line with previous studies that applied alpha shapes to genital form, which also found consistency between complexity scores and informal qualitative observations (Gardiner et al., 2018; Orbach et al., 2021). Indeed, all species in the family Lutrinae are grouped with low PC1 scores, commensurate with their ‘simple’ shape at both coarse and fine scales. This finding is expected given the rod‐like morphology of the group, which has been thought to perhaps facilitate mating in water (Kitchener et al., 2017). All Mustelinae species, such as *M. nudipes*, *M. sibirica* and *Mustela strigidorsa* have high PC1 scores and feature bends, hooks, varying degrees of urethral grooves and pronounced dorsal crests, consistent with qualitative descriptions in Baryshnikov et al. (2003). Alpha shapes PC2 may define aspect ratio, with long slender bacula such as *M. nudipes* and *N. nasua* scoring highly and species with short, thick bacula, best exemplified by the sole member of the family Ailuridae, *A. fulgens,* attaining low scores (Figure 1a).

AriaDNE PC1 shows positive loading for some fine scale morphologies including bends and additional projections at the tip. Again, the family Lutrinae are grouped with low PC1 scores, while two distantly related species of Procyonidae, *N. nasua* and *P. flavus*, have high PC1 scores, and both feature rounded shafts and pronounced heads. Neither of these species feature explicit hooks; however, they do have other unique characteristics, particularly *P. flavus*, which has a symmetrical subdivision at the tip, with bulbous condyles separated by a deep indent (Figure 1b). In line with these results, two Mustela species with extreme bends and urethral grooves, *M. strigidorsa* and *M. nudipes*, have high ariaDNE PC1 results, whereas the other Mustela species are grouped with lower scores. AriaDNE PC2 accounts for 27% of total variation and cannot be confidently ascribed to a single driving morphological feature of the shaft or tip. Although high PC2 scoring species include *P. flavus*, *M. sibirica*, *M. kathiah* and *Eira barbara* all possess some combination of a triangular cross‐section at midshaft, long urethral groove and/or a scoop‐like distal tip. Species with low PC2 scores feature less rugose proximal ends, which may suggest that PC2 is describing some of the less frequently characterised surface morphology of the bacula, including soft‐tissue attachment sites.

PCAs for alpha shapes and ariaDNE show some consistency in grouping of families with similar gross morphologies. This is not surprising given the baculum is commonly used in taxonomic studies (Abramov, 2002; Van Zyll de Jong, 1972). Within Mustelidae there is clustering of closely related subfamilies such as Guloninae, Ictonychinae and Helictidae in both alpha shapes and ariaDNE PCAs. As anticipated by the observable high levels of morphological diversity, the most species‐rich subfamily in the data set, Mustelinae, appear to have higher levels of quantified shape complexity. Yet, scatter is relatively high in both alpha shapes and ariaDNE complexity values. The red panda (*A. fulgens*) possesses low PC1 results for both alpha shapes and ariaDNE analysis with clear separation from their closest subfamily within the data set, Procyonidae (Figure 1). It is now believed that the earliest branching family of Musteloidea is Mephitidae, placing Ailuridae closer to Mustelidae and Procyonidae (Law et al., 2017; Sato et al., 2009). A future analysis of this data set to include Mephitidae bacula would therefore be beneficial to understanding the extent of morphological variation across Musteloidea.

Linear regression models and PGLS were fitted to both alpha shapes and ariaDNE PCA results, with life history variables added to explore evolutionary drivers of shape complexity. A confounding factor in PGLS analyses, and indeed in other phylogenetic comparative studies (De Meester et al., 2019; Todorov et al., 2022), is the estimation of *λ* from a small sized sample. While *λ* can successfully discriminate between complex models of trait evolution, it is negatively influenced when sample size is small and could potentially fall outside of its normal range (0–1), thus becoming unreliable (Münkemüller et al., 2012). One solution is to fix *λ* at 1 (a constant rates model) and explore any changes in outcomes of the regression (Todorov et al., 2022). To promote best practise and explore the effect of sample size upon phylogenetic comparative methods, the regression analyses were conducted here as a phylogenetically uncorrected linear model (*λ* = 0), PGLS (*λ* = maximum likelihood estimate) and PGLS *(λ* = 1). Significant correlations between alpha shapes PC1 and both intromission duration and relative testes mass disappeared when *λ* was fixed at 1, compared to when *λ* was estimated but remained significant between ariaDNE PC1 and intromission duration (Table 2), suggesting these results remain robust in light of sample size and phylogenetic uncertainty. This study should serve as a note of caution for the effects of estimating *λ* for those conducting future comparative phylogenetic analysis on small datasets. Previous studies have used large intraspecific datasets to examine baculum characteristics, including bone weight and length (Özen, 2018), and allometry (Csanády & Onderková, 2018; Miller & Nagorsen, 2008; Schulte‐Hostedde et al., 2011) within the same Musteloid species (including *Martes foina*, *Martes caurina*, *Martes americana*, *Martes pennanti* and *Mustela erminea*). Additionally, many studies use large intraspecific samples for the purpose of taxonomy (*Mustela nivalis*: Abramov & Baryshnikov, 2000) and age (*M. erminea hibernica*: Sleeman, 2004). Here, we focus our sampling effort on digitising a large interspecific data set to test broader evolutionary questions, as opposed to collecting multiple replicates per species. Many of the taxa included herein are infrequently represented in museum collections, and quantifying within‐species variation outside of commonly studied taxa would be challenging. However, future studies would benefit from a greater insight into this within‐species variation, especially if accompanied by individual‐level associated data (body mass, testes mass etc.).

Both alpha shapes PC1 and ariaDNE PC1 were significantly correlated to two proxies of post‐copulatory sexual selection. These results suggest that high shape complexity, as determined by both alpha shapes and ariaDNE, may be driven by increased intromission duration across the group, supporting previous comparative studies in mammals (Brassey et al., 2020; Brindle & Opie, 2016; Dixson et al., 2004). In addition to intromission duration, there has been broad support for the prediction that relative testes mass indicates intensity of sperm competition (Birkhead & Møller, 1996; Simmons & Fitzpatrick, 2012), with a recent quantitative meta‐analysis justifying the use of these assumptions in research (Lüpold et al., 2020). The results presented here provide evidence of a correlation between relative testes mass and baculum shape complexity when quantified using the alpha shapes methodology. This finding contrasts with a previous study, which found no significant correlation between alpha shape complexity and relative testes mass in a wider carnivore group (Brassey et al., 2020). A potential explanation for this is our present focus on the Musteloidea. While Musteloidea possess relatively well developed bacula, the wider order, Carnivora, can vary between extremely large and robust bones in groups such as Ursidae and Canidae (Dixson, 1995) to fragments of bone considered ‘rudimentary’ in the family Felidae (Tumlison & McDaniel, 1984) making regional complexity difficult to quantify and compare.

This study provides one of the first comparisons of alpha shapes and ariaDNE complexity metrics across a whole biological structure (see Supporting Information for all complexity results). While PCA identified broadly similar taxonomic groupings resulting from alpha shape refinement coefficient and ariaDNE bandwidth weightings, the two metrics extracted subtly different morphological features, as the two methods are mathematically distinct and operate on two different forms of 3D data (mesh vs point cloud) for the same 3D object. Alpha shape fits are seemingly more sensitive to coarse morphological features (particularly regions of concavity; Gardiner et al., 2018), which leads to higher scores for species with hooks, bends and triangular shaft shape. In contrast, fine‐scale features including additional projections at the tip and surface detail of the bone score higher in ariaDNE complexity. This is to be expected given previous applications of ariaDNE are primarily found in the field of dental topography (Fulwood et al., 2021; Lang et al., 2021). The present study compares quantitative metrics of shape complexity against qualitative/subjective morphological character scores. By its very nature, character coding is sensitive to interobserver variability (Davis et al., 2013; Kimmerle et al., 2008; Wilczak et al., 2017). Therefore, we conducted a post‐hoc analysis of interobserver error to assess the sensitivity of our qualitative results. A higher level of agreement with the main author's scores (defined as >62%), was found in seven of the nine characters. However, for two characters, ‘Pronounced Head’ and ‘Shape of median portion of the mid‐shaft’, there was much lower agreement between the observers (see details in Supporting Information: Appendix 1). Interestingly, those characters with which we find a significant correlation to the 3D complexity metrics are also consistent between observers, suggesting that the least ambiguous characters are often those to which the algorithms are most sensitive. An interesting avenue of future research would be to explore where agreement between algorithm and observers occurs and why, incorporating the disciplines of psychology and human perception.

Musteloidea were chosen due to their resolved phylogeny and well‐developed bacula (Čanády & Onderková 2016; Flynn et al., 2005). The results presented here suggest that post‐copulatory mechanisms of sexual selection may be exploited by these taxa. However, there is no support for a relationship between pre‐copulatory selection pressure (as measured by SSD) and baculum complexity. Some of the suggested advantages of male‐biased SSD, notably undertaking the energy‐expensive tasks of long‐distance mate searching coupled with volatile and prolonged intromission, are relevant to many species of Musteloidea, especially species within the genus *Mustela* (Krawczyk et al., 2011). However, while previous research suggests the positive allometry between body size and baculum length and/or width implies the baculum is an indicator of male quality (Krawczyk et al., 2011; Miller & Nagorsen, 2008), the results presented here indicate that there is no relationship between male‐biased SSD and baculum shape complexity. This may be for two reasons; firstly that pre‐copulatory sexual selection is simply not driving baculum complexity in the Musteloidea. Indeed, many of the species are solitary and may coincide relatively rarely (Lukas & Clutton‐Brock, 2013). While many of these species exploit polygynous matting patterns (Law & Mehta, 2018), solitary species are territorial, which may lead to overlap promiscuity (Sandell, 1986) or successive polygyny (Lodé, 2001), which perhaps reduces the pressure of pre‐copulatory competition and choice. Second, SSD might not be a good proxy of pre‐copulatory selection in Musteloidea. Evidence has associated SSD with other factors in Musteloidea, including their process of acquiring food (Loy et al., 2004), diet choice (Noonan et al., 2016) and niche partitioning (Law & Mehta, 2018). This, combined with the territorial nature of many of these species (Lodé, 2001; Moors, 1980) may explain the absence of precopulatory male–male conflict.

Our results suggest that Musteloidea are under post‐copulatory sexual selection pressures. This is in line with previous research that found male intrasexual competition and investment in testicular tissue were correlated to shorter mating seasons (Iossa et al., 2008), the use of reproductive mechanisms such as embryonic diapause and delayed implantation (Mead, 1989) and the presence of polyandry, where the female mates with several males during oestrus (Kenagy & Trombulak, 1986). In experimental studies, baculum form has been shown to vary with post‐copulatory pressures. Artificially altered levels of sexual selection led to a significant increase in bacula width (Simmons & Firman, 2013) and baculum size was significantly associated with reproductive success (Stockley et al., 2013), suggesting that intra‐sexual post‐copulatory sexual selection pressure may be driving bacula evolution (Brindle & Opie, 2016).

In addition to this, some musteloid species, such as *Martes martes* (Landowski, 1962), *Neovison vison* (Enders, 1952) and members of the subfamily *Mustelinae* (Carroll et al., 1985; Dixson et al., 2004) have been observed partaking in extremely long periods of intromission of up to 3 h. Behaviour such as mate guarding, whereby male bites the female's neck, physically restricting her from leaving during or immediately following copulation, usually interspersed with short burst of thrusting, has been recorded in some musteloid species (Dewsbury, 1972), including the *Neovision vision* (Fleming, 1996), *Mustela putorius furo* (Hammond & Marshall 1930), *Mustela frenata* (Wright, 1948), *Ictonyx striatus* and *Poecilogale albinucha* (Rowe‐Rowe, 1978). Prolonged intromission duration may serve to decrease the likelihood of insemination by successive males in species whereby multi male matings occur (Dixson, 1995). Studies using invertebrate models have shown this may be due to reduced female receptivity or increased rate of sperm transfer (Garcia‐Gonzalez, 2004; Linn et al., 2007; Parker, 1970).

The results presented here suggest that the presence of a distal hook may act to physically secure the female or otherwise maintain intromission during these long periods. While intromission duration values have been gathered from published sources, they are sparsely reported and there are difficulties in assessing exact timing, especially given that the period of time mounting might not equal the time in which intromission is occurring (Brassey et al., 2020). The ability to non‐invasively quantify male copulatory kinematics, especially with regard thrusting activity and timing of ejaculation, could offer further explanation for the variance in genital form.

While this study provides a comprehensive analysis of musteloid baculum shape there are additional queries to address before a complete assessment of sexual selection mechanisms can be made. This data comprises approximately 38% of extant Musteloidea species and span a wide range of sub‐families, however, with the increasing accessibility of virtual data through internet repositories such as MorphoSource (Boyer et al., 2016) it may be that future analyses can utilise 3D digital versions of rare specimens to ensure more thorough coverage of musteloid species. Additionally, a quantitative analysis of the corresponding female anatomy in these species would prove extremely informative. Historically overlooked due to difficulties in collection and imaging of soft tissue (Orbach, 2022), modern techniques (including silicone casting, computed tomography and magnetic resonance imaging) offer the opportunity for shape complexity analysis at the same standard as those techniques used here (Clear et al., 2022). A comprehensive understanding of both male and female genital anatomy, covariance of genital morphology (André et al., 2020) and analysis of copulatory fit, as conducted in other species (cetaceans: Orbach et al., 2017; caiman: Moore et al., 2021), will provide the opportunity to explore how variation in form might influence copulatory function in Musteloidea.

## CONFLICT OF INTEREST STATEMENT

The authors declare no conflict of interest.

### PEER REVIEW

The peer review history for this article is available at https://www.webofscience.com/api/gateway/wos/peer-review/10.1002/jmor.21572.

## Supporting information

Supporting information.

Supporting information.

Supporting information.

## Data Availability

Shape complexity and life‐history data are available online at 10.6084/m9.figshare.c.6244917.v1.
