# Supplementary material for: Baculum shape complexity correlates to metrics of post‐copulatory sexual selection in Musteloidea
Source: J Morphol. 2023 Mar 1;284(4):e21572. doi: 10.1002/jmor.21572 (PMC10952176; doi:10.1002/jmor.21572)
Supplement: Supplementary file 1 — Supporting information. [file JMOR-284-0-s002.docx]

**Appendix 1**

**Baculum shape complexity correlates to metrics of postcopulatory sexual selection in Musteloidea.**

*Table 1. Interobserver Qualitative Scores (% of independent scores matching those taken by the first author)*

|  | Characters relating to bacula head | | Characters relating to bacula shaft | | Characters relating to bacula tip | | | | |
| --- | --- | --- | --- | --- | --- | --- | --- | --- | --- |
| Species | Pronounced head | Opening on head | Shape of median portion of mid-shaft | Absence of a urethral groove | Abrupt bend in distal tip | Presence of distal hook | Subdivision of distal tip in the ventral plane | Shape of distal tip (symmetry) | Complexity of distal tip (additional projections present) |
| *Ailuris fulgens* | 60 | 100 | 80 | 100 | 100 | 100 | 40 | 80 | 20 |
| *Aonyx cinera* | 20 | 100 | 20 | 100 | 40 | 20 | 80 | 100 | 40 |
| *Gulo gulo* | 60 | 100 | 100 | 80 | 40 | 20 | 40 | 80 | 100 |
| *Lutra lutra* | 40 | 100 | 40 | 20 | 80 | 100 | 100 | 20 | 80 |
| *Martes flavigula* | 20 | 100 | 40 | 20 | 60 | 100 | 40 | 100 | 80 |
| *Meles meles* | 60 | 100 | 80 | 40 | 80 | 100 | 100 | 60 | 80 |
| *Mellivora capensis* | 80 | 100 | 20 | 60 | 80 | 60 | 20 | 80 | 60 |
| *Mustela lutreola* | 20 | 100 | 40 | 40 | 60 | 100 | 100 | 60 | 100 |
| *Mustela nigripes* | 40 | 100 | 20 | 60 | 60 | 100 | 100 | 100 | 100 |
| *Mustela sibirica* | 60 | 100 | 40 | 100 | 80 | 80 | 100 | 60 | 100 |
| **Mean** | **46** | **100** | **48** | **62** | **68** | **78** | **72** | **74** | **76** |

The qualitative scoring method was checked for interobserver error by having five independent researchers repeat the scoring process on a randomly selected sample of ten Musteloidea bacula samples.

**Results and Discussion**

A higher level of agreement with the main author’s scores (defined as >62%), was found in seven of the nine characters (Opening on head – 100%, Absence of urethral groove – 62%, Abrupt bend in distal tip – 68%, Presence of distal hook – 78%, Subdivision of distal tip – 72%, Shape of distal tip – 74% and Complexity of distal tip – 76%). Two characters ‘Pronounced Head’ and ‘Shape of median portion of the mid-shaft’ achieved a 46% and 48% match with the main authors, respectively.

Both Pronounced Head’ and ‘Shape of median portion’ were based on the degree of difference between two shapes (a gradually larger head and a gradually more circular mid-shaft), neither sought to identify the absolute presence or absence of a defined feature (like a hook, foramen or urethral groove). Where categories are not clearly discernible we might expect to find less agreement between assessors and lower interobserver reliability.
